# Galectin-3 Interacts with Vascular Cell Adhesion Molecule-1 to Increase Cardiovascular Mortality in Hemodialysis Patients

**DOI:** 10.3390/jcm7100300

**Published:** 2018-09-24

**Authors:** Wen-Chin Ko, Cheuk-Sing Choy, Wei-Ning Lin, Shu-Wei Chang, Jian-Chiun Liou, Tao-Hsin Tung, Chih-Yu Hsieh, Jia-Feng Chang

**Affiliations:** 1 College of Medicine, Fu Jen Catholic University, New Taipei City 242, Taiwan; 086938@mail.fju.edu.tw; 2 Division of Cardiology, Department of Internal Medicine, Cathay General Hospital, Taipei 106, Taiwan; 3 Department of Nursing, Yuanpei University of Medical Technology, Hsinchu 300, Taiwan; prof.choy@gmail.com; 4 Department of Community Medicine, En Chu Kong Hospital, New Taipei City 237, Taiwan; 5 Renal Care Joint Foundation, New Taipei City 220, Taiwan; fish37435@Hotmail.com; 6 Graduate Institution of Biomedical and Pharmaceutical Science, College of Medicine, Fu Jen Catholic University, New Taipei City 242, Taiwan; 081551@mail.fju.edu.tw; 7 Department of Civil Engineering, National Taiwan University, Taipei 106, Taiwan; changsw@ntu.edu.tw; 8 School of Biomedical Engineering, Taipei Medical University, Taipei 110, Taiwan; jcliou@tmu.edu.tw; 9 Department of Medical Research and Education, Cheng Hsin General Hospital, Taipei 112, Taiwan; ch2876@gmail.com; 10 Department of Internal Medicine, En Chu Kong Hospital, New Taipei City 237, Taiwan; 11 Ph.D. Program in Nutrition and Food Science, College of Human Ecology, Fu Jen Catholic University, New Taipei City 242, Taiwan

**Keywords:** galectin, cell adhesion molecules, mortality, hemodialysis

## Abstract

Background: Interactions and joint effects of galectin-3 and vascular cell adhesion molecule 1 (VCAM-1) on risks of all-cause and cardiovascular (CV) mortality remain unclear in patients with maintenance hemodialysis (MHD). Methods: Unadjusted and adjusted hazard ratios (aHRs) of mortality risks were analyzed between higher and lower concentration groups of serum galectin-3 and VCAM-1. The modification effect between serum galectin-3 and VCAM-1 on mortality risk was investigated using an interaction product term. Results: During follow-up, galectin-3 and VCAM-1 were associated with incremental risks of all-cause mortality (aHR: 1.038 (95% confidence interval (CI): 1.001–1.077) and 1.002 (95% CI: 1.001–1.003), respectively). Nonetheless, VCAM-1 but not galectin-3 predicted CV mortality (aHR: 1.043 (95% CI: 0.993–1.096) and 1.002 (95% CI: 1.001–1.003), respectively). In the interaction analysis, patients with combined higher galectin-3 (>29.5 ng/mL) and VCAM-1 (>1546.9 ng/mL) were at the greatest risk of all-cause and CV mortality (aHR: 4.6 (95% CI: 1.6–13.4), and 4.2 (95% CI: 1.3–14.4), respectively). The interactions between galectin-3 and VCAM-1 with respect to all-cause and CV mortality were statistically significant (*p* < 0.01 and < 0.05, respectively). Conclusion: Galectin-3 and VCAM-1 could serve as a promising dual biomarker for prognostic assessment, considering their joint effects on pathogenesis of leukocyte trafficking and atherothrombosis.

## 1. Introduction

Galectin-3 is a versatile protein involved in pathogenesis of diverse human diseases, including complex types of cancer, sepsis, cardiovascular (CV) disease, brain injury, pulmonary disorders, fibrosis in different organs, chronic inflammation and autoimmune rheumatic diseases [1,2,3]. In light of the multifaceted roles of galectin-3, identifying a clear etiology is difficult once the serum concentration of galectin-3 increases. Given that CV disease remains the leading cause of morbidity and mortality in patients on maintenance hemodialysis (MHD) [4], searching for optimal biomarkers as early warning signs of CV events is warranted. Our previous study demonstrates that serum concentration of vascular cell adhesion molecule 1 (VCAM-1) is a strong independent predictor of CV mortality in MHD patients [5]. Moreover, galectin-3 acts as an amplifier of inflammation in atherosclerotic plaque progression through macrophage activation and monocyte chemoattraction, interacting with VCAM-1 in leukocyte trafficking and adhesion in basic research [6,7]. Thus we aimed to explore the joint effect of serum concentrations of galectin-3 and VCAM-1 on all-cause and CV mortality in MHD patients. The modification effect between galectin-3 and VCAM-1 on mortality risks was also investigated in this population-based study.

## 2. Methods 

### 2.1. Cohort

The investigation was conducted according to the study method previously described [5]. Patients undergoing MHD treatment for at least 3 months were eligible for inclusion. All patients had to be older than 18 years of age and be receiving thrice-weekly MHD. Patients were excluded from the study if they had severe cardiac failure (New York Heart Association class IV), terminal illness, active infections, active malignancy, protein-energy wasting, incomplete data or were unwilling to participate (Supplemental Figure S1). Hemodialysis (HD) vintage was defined as the duration of time between the first day of HD treatment and the first day that the patient entered the cohort. Blood pressure was recorded in the horizontal recumbent position before dialysis session. Pre-dialysis blood samples were obtained from the existing vascular access. The extension of the study period was approved by the Research Ethics Review Committee of the En Chu Kong Hospital (ECKIRB1070102).

### 2.2. Bio-Demographic and Biochemical Parameters

The following bio-demographic and laboratory parameters of each patient were recorded at baseline: age, gender, hypertension, diabetes mellitus (DM), HD vintage, smoking history, blood pressure, potassium, calcium, phosphorus, creatinine, pre-dialysis blood urea nitrogen, alanine aminotransferase, fasting glucose, albumin, C-reactive protein (CRP), uric acid, normalized protein catabolic rate (nPCR), total cholesterol, triglyceride, low-density lipoprotein, iron profiles, hemoglobin, intact parathyroid hormone, galectin-3 and VCAM-1. We adjusted serum calcium according to the following equation: adjusted calcium = measured calcium+ ((4.0 − serum albumin in g/dL) × 0.8). Serum concentrations of galectin-3 and VCAM-1 were measured by a commercial quantitative enzyme-linked immunosorbent assay and all laboratory tests were performed by standard procedures with certified methods.

### 2.3. Outcomes and Follow-up

CV mortality in study patients was defined as death attributable to myocardial ischemia and infarction, heart failure, fatal arrhythmia, cardiac arrest because of other causes, cerebrovascular diseases, pulmonary embolism, peripheral artery diseases and sudden otherwise unexplained death. Non-CV mortality was defined as all other causes of death, i.e. infection, malignancies, gastrointestinal hemorrhage, accidents and miscellaneous. All-cause mortality included CV and non-CV death. Patients were censored at last follow-up, switched to another dialysis unit or received a kidney transplant.

### 2.4. Statistical Methods

Continuous variables were presented as mean ± standard deviation, and categorical variables were expressed as number (%). The correlation coefficients between covariates of interest were calculated. The univariate Cox regression analysis was performed to investigate the independence of risk factors associated with all-cause and CV mortality. The included subjects for final analysis were further stratified into higher and lower concentration groups by median values of galectin-3 and VCAM-1, respectively. Unadjusted and multivariable adjusted hazard ratios (aHRs) of mortality risks were calculated for different categories of serum galectin-3 and VCAM-1 in the Cox regression model, including galectin-3, VCAM-1, albumin, age, CRP, nPCR, and smoking. The modification effect between serum galectin-3 and VCAM-1 on mortality risks was determined using an interaction product term. According to methods previously described, an interaction occurs when the impact of a risk factor on outcome is changed by the value of a third variable, sometimes referred to as effect modification [8,9].

The multiplicative interaction term derived from the product of galectin-3 and VCAM-1 acts as a third variable. We evaluated if the effect of galectin-3 on mortality risks was modified by VCAM-1 through incorporating an interaction term in the multivariate model. The cumulative survival probability and proportional hazards were presented by graphical methods. A significant product term indicates that there is an interaction between galectin-3 and VCAM-1 on the probability of mortality risk. A *p* value < 0.05 was considered statistically significant. We used PASW Statistics SPSS18 to analyze all bio-clinical data of MHD patients. 

## 3. Results

The final study sample included 86 MHD patients with complete medical records and follow-up. Baseline bio-clinical data of the whole study population with comparison between survivors and non-survivors are summarized in Table 1. The mean age was 60.0 ± 12.6 years; approximately 45% were male. Prevalences of DM, hypertension, and smoking were 40.7%, 45.3%, and 22.1%, respectively. The mean duration of follow-up was 53.3 ± 6.2 months. The overall mortality rate was 40.7% during follow-up, corresponding to an annual mortality rate of 9.2%. Nineteen patients (54.3%) died from CV causes, and 16 (45.7%) non-CV deaths occurred. The bio-clinical parameters that differed significantly between survivors and non-survivors included age, smoking, albumin, nPCR, CRP, galectin-3, and VCAM-1.

Table 2 summarizes the bivariate correlation coefficients between prognostic factors (galectin-3 and VCAM-1) and bio-clinical parameters of interest in MHD patients at baseline. Galectin-3 was significantly correlated with VCAM-1 (*r* = 0.49; *p* < 0.01), nPCR (*r* = −0.31; *p* < 0.01), and CRP (*r* = 0.32; *p* < 0.01), respectively. VCAM-1 was in turn significantly correlated with smoking (*r* = 0.26; *p* < 0.05), HD vintage (*r* = 0.22; *p* < 0.05), nPCR (*r* = −0.23; *p* < 0.05), CRP (*r* = 0.24; *p* < 0.05), and albumin (*r* = −0.21; *p* < 0.05). In the univariate Cox regression analysis of prognostic factors, galectin-3, VCAM-1, albumin, age, CRP, and nPCR were significantly associated with all-cause mortality. Furthermore, galectin-3, VCAM-1, age, CRP nPCR, and smoking were significantly associated with CV mortality. The univariate Cox regression analysis of prognostic factors unveiled that galectin-3, VCAM-1, nPCR, age, and CRP were all significantly associated with all-cause and CV mortality. The multivariate Cox regression model demonstrated VCAM-1, age and CRP were still significantly associated with all-cause and CV mortality. However, galectin-3 predicted all-cause death rather than CV mortality after multivariate adjustment (Table 3 and Table 4).

### 3.1. Galectin-3

**Figure 1:**
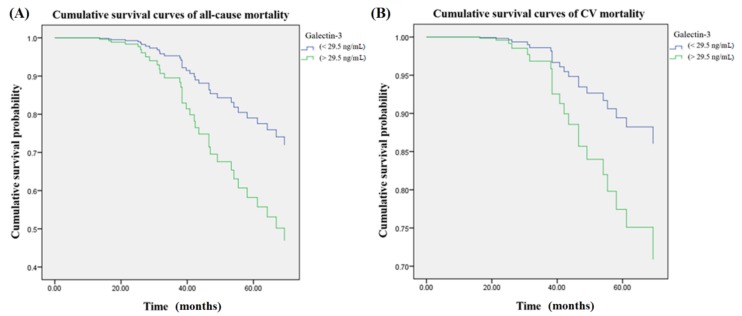
Cumulative survival curves of mortality risks with respect to serum concentrations of galectin-3 after adjusting for galectin-3, VCAM-1, albumin, age, CRP, nPCR, and smoking during follow-up. (**A**) There was an association between higher concentration group of galectin-3 (>29.5 ng/mL) was associated with an incremental risk of all-cause mortality. (**B**) The association between higher galectin-3 concentrations and cardiovascular (CV) mortality was insignificant after multivariable adjustment. VCAM-1 = vascular cell adhesion molecule 1; CRP = C-reactive protein; nPCR = normalized protein catabolic rate.

Figure 1 illustrated cumulative survival curves of all-cause and CV mortality with respect to higher and lower categories of galectin-3 after multivariate adjustment in the Cox regression model. Multivariable-adjusted results demonstrated higher galectin-3 levels were associated with incremental risks for all-cause mortality (aHR: 2.3 (95% CI: 1.1–4.9)) instead of CV mortality (aHR: 2.3 (95% CI: 0.8–6.2)).

### 3.2. VCAM-1

**Figure 2:**
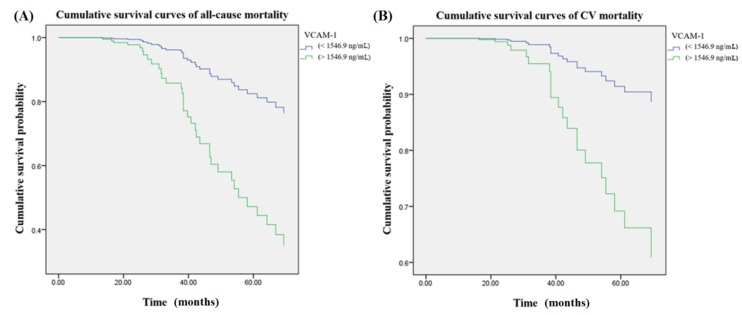
Cumulative survival curves of mortality risks with respect to serum concentrations of VCAM-1 after adjusting for galectin-3, VCAM-1, albumin, age, CRP, nPCR, and smoking during follow-up. (**A**) There was an association between higher VCAM-1 concentrations (>1546.9 ng/mL) and incremental risk of all-cause mortality. (**B**) The association between higher VCAM-1 concentrations and CV mortality remained robust after multivariable adjustment. VCAM-1 = vascular cell adhesion molecule 1; CRP = C-reactive protein; nPCR = normalized protein catabolic rate.

Figure 2 illustrated cumulative survival curves of all-cause and CV mortality with respect to higher and lower categories of VCAM-1 after multivariate adjustment in the Cox regression model. Multivariable-adjusted results demonstrated higher VCAM-1 levels were associated with incremental risks for all-cause mortality (aHR: 3.9 (95% CI: 1.8–8.3)) and CV mortality (aHR: 4.1 (95% CI: 1.5–11.3)), respectively.

### 3.3. Interaction Analysis between Galectin-3 and VCAM-1 on Mortality Risks

**Figure 3:**
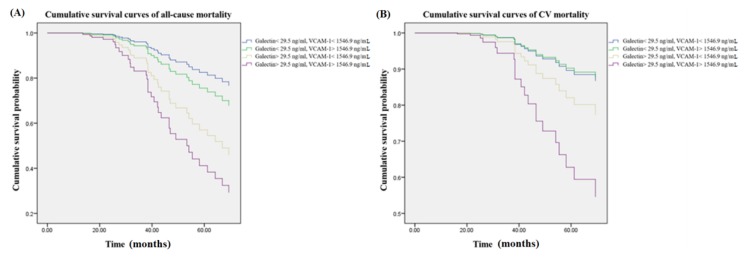
Cumulative survival curves of mortality risks with respect to different concentration groups of galectin-3 and VCAM-1 after adjusting for galectin-3, VCAM-1, albumin, age, CRP, nPCR, and smoking during follow-up. (**A**) The group with combined higher concentrations of galectin-3 (>29.5 ng/mL) and VCAM-1 (>1546.9 ng/mL) showed higher risks for all-cause mortality as compared to the reference group (galectin-3 < 29.5 ng/mL and VCAM-1 < 1546.9 ng/mL). (**B**) The group with combined higher concentrations of galectin-3 (>29.5 ng/mL) and VCAM-1 (>1546.9 ng/mL) showed greater CV mortality risks as compared to the reference group (galectin-3 < 29.5 ng/mL and VCAM-1 < 1546.9 ng/mL). The interaction analysis between serum galectin-3 and VCAM-1 remained statistically significant for all-cause mortality (*p* < 0.01) and CV mortality (*p* = 0.039), respectively. VCAM-1 = vascular cell adhesion molecule 1; CRP = C-reactive protein; nPCR = normalized protein catabolic rate.

Figure 3 illustrated patients with higher galectin-3 (>29.5 ng/mL) and VCAM-1 (>1546.9 ng/mL) had the greatest mortality risks for not only all-cause mortality (aHR: 4.6 (95% CI: 1.6–13.4)) but also CV mortality (aHR: 4.2 (95% CI: 1.3–14.4)) compared to the reference group (galectin-3 < 29.5 ng/mL and VCAM-1 < 1546.9 ng/mL) in the multivariate Cox regression model. The interaction analysis between serum galectin-3 and VCAM-1 was statistically significant for all-cause mortality (*p* < 0.01) and CV mortality (*p* = 0.039), respectively. 

## 4. Discussion

We show a brand new idea that adding VCAM-1 to galectin-3 provides more robust predictive values for not only all-cause but also CV mortality in this prospective cohort study. In more depth, higher plasma concentrations of galectin-3 and sVCAM-1 interact to increase the risk of atherosclerotic CV events. Several important findings in this work deserve further discussion. 

Mechanistically, myocardial stretch induced upregulation of galectin-3 and downstream connective tissue growth factor [10], which acted as a profibrotic and proinflammatory factor in our previous studies [11,12]. Furthermore, galectin-3 regulates many biological functions and signaling pathways with regard to cell proliferation, migration, adhesion, and cell cell interactions through binding with galactose-containing glycoproteins on the cell surface [13]. Accordingly, plasma levels of galectin-3 may reflect underlying mechanisms involved in leukocyte adhesion to the vascular endothelium and atherosclerotic plaque progression [14]. Drechsler et al reported galectin-3 concentrations increased with renal function deterioration and independently predicted all-cause mortality, myocardial infarction, stroke, and death due to infection in patients with renal impairment [15]. Nonetheless, another study demonstrated galectin-3 did not independently predict recurrent CV events in patients with established coronary heart disease after adjusting markers of hemodynamic stress, myocardial injury, inflammation, and renal impairment [16]. Collectively, current evidence does not support single use of galectin-3 to evaluate the prognosis in patients with heart failure [17]. It is suggested that galectin-3 should be added to troponins and natriuretic neuropeptides for CV prognostic assessment in high-risk population [18].

Why is galectin-3 not sufficiently specific as a biomarker of CV mortality risk? Ho et al reported that galectin-3 seemed to have a stronger association with non-CV death from than CV causes [19]. Galectin-3 is common to inflammatory diseases and up-regulated in diverse fibrotic processes, including the heart, lung, liver, and kidney [3]. This lack of tissue specificity is likely to have limitations for consideration of galectin-3 in routine screening policies [20].

Does galectin-3 interact with VCAM-1 to increase the risk of CV mortality? Our previous data demonstrate that serum concentration of VCAM-1 is a promising biomarker to predict all-cause and CV mortality in MHD patients [5]. Given that both galectin-3 and VCAM-1 were intricately involved in leukocyte trafficking and atherosclerotic plague progression, we assumed that the risk of CV mortality would be augmented among patients with higher levels of galectin-3 and VCAM-1. In this study, the modification effect between VCAM-1 and galectin-3 on all-cause and CV mortality was examined using an interaction product term according to previous methods of moderation analysis [8,9]. As expected, the effects of galectin-3 on all-cause and CV mortality risks were modified by VCAM-1 in the multivariate model. Our data show that patients with higher serum concentrations of galectin-3 (>29.5 ng/mL) and VCAM-1 (>1546.9 ng/mL) have the greatest risk of all-cause and CV death. Furthermore, the effects of higher VCAM-1 on mortality are abated by lower levels of galectin-3, and vice versa. The interaction effect between galectin-3 and VCAM-1 on all-cause and CV mortality was statistically significant (*p* < 0.01 and 0.05 for the interaction term, respectively). Galectin-3 handling and VCAM-1-driven pathways overlap in leukocyte trafficking and atherosclerosis, suggesting galectin-3 and VCAM-1 may share common pathogenesis in CV mortality (Supplemental Figure S2).

Our study has several limitations. First, cross-sectional laboratory values might not reflect substantial intra-individual variability over time. Second, our sample size is small, limiting the statistical power in the multivariate adjustment and threshold selection. Third, serum concentrations of galectin-3 and VCAM-1 were measured from frozen rather than from fresh samples. Finally, prospective nonrandomized analysis is subject to residual confounders. 

## 5. Conclusions

Early detection of high-risk patients undergoing MHD is important. Galectin-3 and VCAM-1 predict all-cause death in MHD patients, and the combination test provides more robust predictive values for not only all-cause but also CV mortality. While considering joint effects of galectin-3 and VCAM-1 on pathogenesis of leukocyte trafficking and atherothrombosis, galectin-3 and VCAM-1 could serve as promising dual biomarker for prognostic assessment. 

## Figures and Tables

**Table 1:** Bio-clinical data of the whole study population with comparison between survivors and non-survivors.

Variables	Overall (*n* = 86)	Survivors (*n* = 51)	Deceased (*n* = 35)
Age (years)	59.9 ± 14.0	52.6 ± 13.1	67.5 ± 8.6
Male, *n* (%)	38 (44.2)	23 (45.1)	15 (42.9)
Diabetes mellitus, *n* (%)	35 (40.7)	19 (37.3)	16 (45.7)
Hypertension, *n* (%)	39 (45.3)	22 (43.1)	17 (48.6)
Smoking, *n* (%)	19 (22.1)	5 (9.8)	14 (40.0)
Systolic blood pressure (mmHg)	135 ± 25	137 ± 25	129 ± 22
Diastolic blood pressure (mmHg)	76 ± 12	78 ± 13	72 ± 11
Hemodialysis vintage (months)	61.2 ± 53.2	58.9 ± 51.6	64.5 ± 56.1
Alanine aminotransferase (IU/L)	13.3 ± 10.2	15.5 ± 11.9	11.1 ± 6.2
Albumin (g/dL)	3.9 ± 0.5	4.0 ± 0.3	3.6 ± 0.5
Total cholesterol (mg/dL)	194.0 ± 49.3	195.2 ± 51.3	192.3 ± 47.1
Triglyceride (mg/dL)	210.0 ± 179.0	201.8 ± 170.5	212.0 ± 193.1
Creatinine (mg/dL)	10.3 ± 1.7	10.6 ± 1.8	9.9 ± 1.8
Uric acid (mg/dL)	7.3 ± 1.3	7.5 ± 1.4	7.1 ± 1.1
Normalized protein catabolic rate (g/kg/day)	1.1 ± 0.3	1.2 ± 0.3	0.9 ± 0.3
Potassium (mmol/L)	4.6 ± 0.9	4.6 ± 0.8	4.6 ± 1.0
Adjusted calcium (mg/dL)	9.2 ± 0.7	9.3 ± 0.7	9.1 ± 0.7
Phosphate (mg/dL)	4.4 ± 1.6	4.6 ± 1.7	4.3 ± 1.3
Intact parathyroid hormone (pg/mL)	150.9 ± 192.7	235.0 ± 32.9	98.3 ± 16.6
Iron (μg/dL)	79.4 ± 33.0	81.5 ± 34.7	74.4 ± 29.4
Total iron binding capacity (μg/dL)	230.0 ± 39.7	236.9 ± 40.6	226.1 ± 37.3
Ferritin (ng/mL)	623.0 ± 317.7	579.5 ± 286.7	665.1 ± 353.1
Hemoglobin (g/dL)	11.0 ± 1.0	10.7 ± 1.3	10.4 ± 1.5
C-reactive protein (mg/dL)	0.7 ± 1.0	0.6 ± 0.5	1.1 ± 1.2
Galectin-3 (ng/mL)	29.5 ± 10.3	25.5 ± 9.6	35.2 ± 8.6
Vascular cell adhesion molecule 1 (ng/mL)	1546.9 ± 331.8	1458.6 ± 303.7	1675.9 ± 332.8

Continuous variables were expressed as mean ± SD. Categorical variables are expressed as *n* (%). Boldface indicates where the values differ significantly between survivors and non-survivors.

**Table 2:** The correlation analysis in galectin-3, VCAM-1, and clinical parameters of interest.

	Galectin-3	VCAM-1
Galectin-3	1.00	0.49 **
Vascular cell adhesion molecule 1	0.49 **	1.00
Age	0.06	0.05
Hemodialysis vintage	0.07	0.22 *
Normalized protein catabolic rate	−0.31 **	−0.212
Albumin	−0.13	−0.21 *
C-reactive protein	0.32 **	0.24 *
Creatinine	−0.14	−0.12
Total cholesterol	−0.18	−0.19
Triglyceride	−0.08	−0.17
Low-density lipoprotein	−0.16	0.17
Uric acid	0.03	−0.08
Potassium	−0.10	−0.15
Glucose	0.03	−0.08
Adjusted calcium	0.02	−0.15
Phosphate	−0.17	−0.01
Hemoglobin	−0.12	−0.25 *
Smoking	0.18	0.26 *

* 0.01 < *p* < 0.05, ** *p* < 0.01.

**Table 3:** Univariate and multivariable Cox regression analysis of prognostic factors for all-cause mortality.

	Cox Univariate		Cox Multivariate	
	HR (95% CI)	*p*-Value	HR (95% CI)	*p*-Value
Galectin-3	1.058 (1.031–1.085)	*p* < 0.01	1.038 (1.001–1.077)	*p* < 0.05
VCAM-1	1.002 (1.001–1.003)	*p* < 0.01	1.002 (1.001–1.003)	*p* < 0.01
Age	1.062 (1.031–1.094)	*p* < 0.01	1.068 (1.031–1.106)	*p* < 0.01
CRP	1.997 (1.487–2.681)	*p* < 0.01	1.686 (1.127–2.522)	*p* < 0.05
Albumin	0.423 (0.217–0.823)	*p* < 0.05	1.701 (0.627–4.611)	*p* > 0.05
nPCR	0.081 (0.022–0.295)	*p* < 0.01	0.158 (0.036–0.698)	*p* < 0.05
Smoking	2.813 (0.813–9.727)	*p* > 0.05	2.360 (0.860–6.475)	*p* > 0.05

VCAM-1 = vascular cell adhesion molecule 1; CRP = C-reactive protein; nPCR = normalized protein catabolic rate; HR = hazard ratio; CI = confidence interval.

**Table 4:** Univariate and multivariable Cox regression analysis of prognostic factors for CV mortality.

	Cox Univariate		Cox Multivariate	
	HR (95% CI)	*p*-Value	HR (95% CI)	*p*-Value
Galectin-3	1.061 (1.028–1.096)	*p* < 0.01	1.043 (0.993–1.096)	*p* > 0.05
VCAM-1	1.002 (1.001–1.003)	*p* < 0.01	1.002 (1.001–1.003)	*p* < 0.05
Age	1.116 (1.056–1.180)	*p* < 0.01	1.126 (1.059–1.196)	*p* < 0.01
CRP	2.200 (1.512–3.202)	*p* < 0.01	2.612 (1.324–5.151)	*p* < 0.01
Albumin	0.480 (0.199–1.159)	*p* > 0.05	4.599 (0.878–14.10)	*p* > 0.05
nPCR	0.171 (0.035–0.841)	*p* < 0.05	0.628 (0.088–4.469)	*p* > 0.05
Smoking	3.383 (1.386–8.261)	*p* < 0.01	1.559 (0.346–7.035)	*p* > 0.05

VCAM-1 = vascular cell adhesion molecule 1; CRP = C-reactive protein; nPCR = normalized protein catabolic rate; HR = hazard ratio; CI = confidence interval.

## Data Availability

The numeric data used to support the findings of this study are available from the corresponding author upon request. Corresponding author Jia-Feng Chang email: 01508@km.eck.org.tw.

## Supplementary Materials

The following are available online at http://www.mdpi.com/2077-0383/7/10/300/s1, Figure S1: Flowchart of patient enrollment, Figure S2: Schematic of joint effects of galectin-3 and VCAM-1 on pathogenesis of leukocyte trafficking and atherothrombosis. Atherosclerotic plaque formation involves enhanced expression of various adhesion molecules on endothelial cells, including ICAM-1 and VCAM-1. Galectin-3 on the cell surface augments attachment between leukocytes and endothelial cells. Fatty streaks are the first signs of atherosclerosis that consist of lipid-containing foam cells in the arterial wall just beneath the endothelium. The mass of the atheroma is composed of a mixture of lipid and inflammatory cells, leading to progressive spatial occupation of arterial lumen. Once a plaque ruptures, the subsequent thromboembolic event increases the risk of cardiovascular mortality. ICAM-1, intercellular adhesion molecule-1. VCAM-1, vascular cell adhesion molecule-1.
