# Supplementary material for: Galectin-3 Interacts with Vascular Cell Adhesion Molecule-1 to Increase Cardiovascular Mortality in Hemodialysis Patients
Source: J Clin Med. 2018 Sep 24;7(10):300. doi: 10.3390/jcm7100300 (PMC6213523; doi:10.3390/jcm7100300)
Supplement: Supplementary file 1 [file jcm-07-00300-s001.pdf]

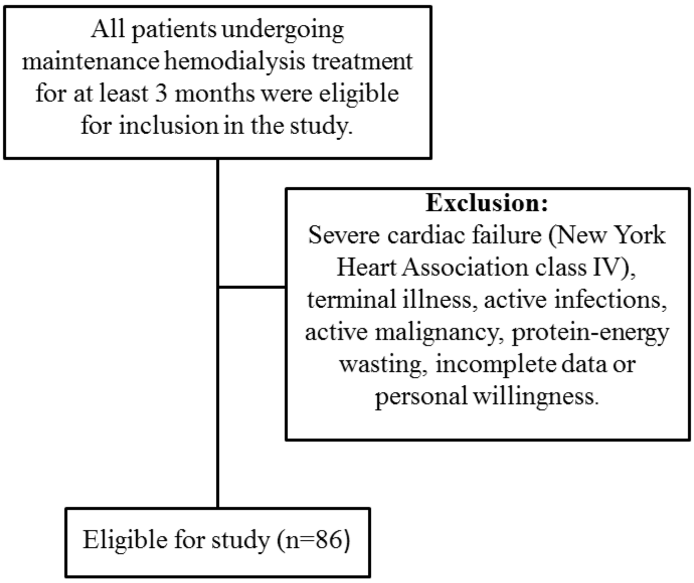

Supplemental Figure 1. Flowchart of patient enrollment.

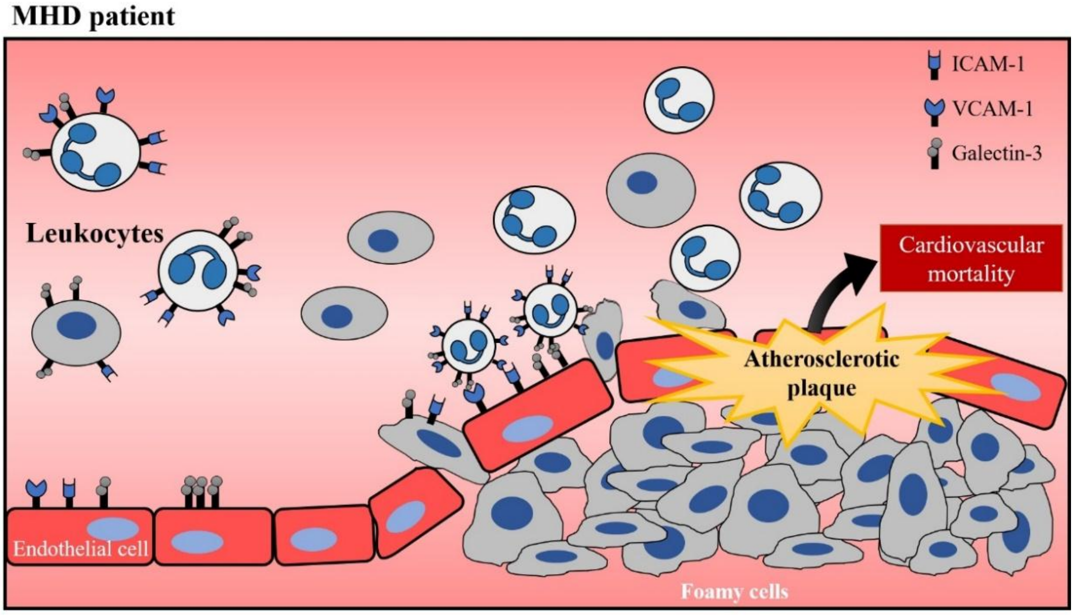

Supplemental Figure 2. Schematic of joint effects of galectin-3 and VCAM-1 on pathogenesis of leukocyte trafficking and atherothrombosis. Atherosclerotic plaque formation involves enhanced expression of various adhesion molecules on endothelial cells, including ICAM-1 and VCAM-1. Galectin-3 on the cell surface augments attachment between leukocytes and endothelial cells. Fatty streaks are the first signs of atherosclerosis that consist of lipid-containing foam cells in the arterial wall just beneath the endothelium. The mass of the "atheroma" is composed of a mixture of lipid and inflammatory cells, leading to progressive spatial occupation of arterial lumen. Once a plaque ruptures, the subsequent thromboembolic event increases the risk of cardiovascular mortality. ICAM-1, intercellular adhesion molecule-1. VCAM-1, vascular cell adhesion molecule-1.
